# The TWEAK–HOIP–HuR Axis: A Novel Mechanism of AMPK Inactivation and Metabolic Reprogramming in Lupus Nephritis Mesangial Cells Hyperproliferation

**DOI:** 10.1155/mi/4149395

**Published:** 2026-03-02

**Authors:** Zongnan Ding, Enzhuo Liu, Xinyue Li, Yujie Zhang, Lei Li, Chenghua Weng, Zhiqin Liu, Zhichun Liu

**Affiliations:** ^1^ Department of Rheumatology and Immunology, The Second Affiliated Hospital of Soochow University, Suzhou, China, suda.edu.cn; ^2^ The College of Food Science and Biology, Hebei University of Science and Technology, Shijiazhuang, China, hebust.edu.cn

## Abstract

Cellular proliferation is intrinsically coupled to metabolic reprogramming, a conserved biological association that modulates the progression of diverse proliferative pathologies. In the present study, we unveil a novel mechanistic link between tumor necrosis factor‐like weak inducer of apoptosis (TWEAK) signaling and Human antigen R (HuR)‐driven metabolic alterations in lupus nephritis (LN), a progressive renal disorder characterized by mesangial cells (MCs) hyperproliferation and glomerular dysfunction. We demonstrate that TWEAK stimulation elicits linear ubiquitination of AMP‐activated protein kinase (AMPK)—a master regulator of cellular energy metabolism—mediated by the E3 ubiquitin ligase HOIL‐1‐interacting protein (HOIP). This posttranslational modification directly results in the functional inactivation of AMPK. Notably, suppressed AMPK activity drives the nuclear export of HuR, a ubiquitously expressed RNA‐binding protein, leading to its subsequent accumulation in the cytoplasmic compartment. In the cytosol, HuR selectively binds to and stabilizes the mRNAs of key cell cycle regulators, with Cyclin D1 being a primary target. This stabilization of Cyclin D1 mRNA ultimately promotes aberrant MCs proliferation, a pathological hallmark of LN. To validate the functional relevance of the pathogenesis, we performed HOIP knockdown experiments in MCs. Consistent with our mechanistic model, HOIP depletion significantly restored AMPK phosphorylation (a well‐established surrogate marker of AMPK activation), suppressed HuR cytoplasmic shuttling, reduced Cyclin D1 expression at both the transcriptional and translational levels, and ultimately inhibited aberrant MCs proliferation in vitro. Collectively, our results establish the TWEAK–HOIP–AMPK–HuR axis as a critical signaling axis that couples metabolic dysfunction to dysregulated cell cycle progression in LN. This work not only provides critical new mechanistic insights into the pathogenesis of mesangial hyperproliferation in LN but also highlights potential therapeutic targets, including HOIP and cytoplasmic HuR, for the development of targeted treatments for LN and other renal diseases characterized by abnormal MCs proliferation.

## 1. Introduction

Systemic lupus erythematosus (SLE) is a chronic, multisystem autoimmune disorder characterized by dysregulated immune responses that lead to pathogenic autoantibody production and immune complex deposition, resulting in widespread tissue and organ damage [1]. Renal involvement, termed lupus nephritis (LN), represents one of the most severe clinical manifestations of SLE, with histopathological evidence present in nearly all renal biopsies from SLE patients [2]. Despite advances in treatment that have improved renal outcomes, a substantial number of LN patients still progress to end‐stage renal disease, underscoring the urgent need for deeper mechanistic insights into disease progression [3, 4].

Although immune dysregulation is central to SLE pathogenesis, a key driver of renal injury in LN is the hyperproliferation of glomerular mesangial cells (MCs). This proliferative response arises from multifactorial mechanisms, including autoantibody‐mediated injury, cytokine signaling, and DNA damage responses [5]. Inflammatory cytokines such as TNF‐α, IL‐6, and tumor necrosis factor‐like weak inducer of apoptosis (TWEAK) potently stimulate MCs’ proliferation through paracrine and autocrine actions [6–8]. Under physiological conditions, MCs help maintain glomerular structure, modulate filtration, and contribute to immune homeostasis [9]. Notably, both TWEAK and its receptor Fn14 are significantly upregulated in the glomerular mesangium in murine lupus models, where their expression correlates with the severity of fibrosis [10]—a finding supported by elevated serum TWEAK levels in LN patients [11]. Importantly, anti‐TWEAK antibody treatment attenuates renal fibrosis in lupus‐prone mice [12], highlighting the TWEAK/Fn14 axis as a key mediator of aberrant cell cycle progression and inflammatory cytokine production [13]. However, the precise mechanisms through which TWEAK promotes MCs proliferation remain incompletely defined.

Cellular proliferation is intimately linked to metabolic reprogramming [14]. We have previously shown that TWEAK downregulates PGC1α, leading to disrupted lipid metabolism and glomerular fibrosis in lupus models [12]. Functioning as a master sensor and effector of cellular energy status, AMP‐activated protein kinase (AMPK) is also a pivotal upstream regulator of PGC1α. It enhances mitochondrial biogenesis and maintains energy homeostasis through a mechanism involving the direct phosphorylation of the PGC1α α‐subunit at specific sites, including Ser538 [15–17]. Relevant to LN, AMPK activation has been shown to suppress high glucose‐induced MCs hyperproliferation [18] and inhibit inflammatory cytokine production in murine lupus models [19]. AMPK activity is regulated through multiple mechanisms, including phosphorylation, allosteric modulation, and ubiquitination [20, 21]. For instance, K63‐linked ubiquitination inhibits AMPK activation by interfering with upstream kinase binding [22].

Human antigen R (HuR), an RNA‐binding protein, posttranscriptionally regulates gene expression by binding to AU‐rich elements (AREs) in the 3^′^ untranslated regions (3’UTR) of target mRNAs, influencing their stability, translation, and localization [23]. Its function is critically dependent on nucleocytoplasmic shuttling, controlled by phosphorylation‐dependent regulation of nuclear localization and export signals [24, 25]. For instance, HuR stabilizes key cell‐cycle transcripts such as cyclin D1 by binding to ARE motifs in its 3^′^UTR, thereby promoting cell cycle progression [26, 27]. TRPM7/PKCα axis induces the nucleocytoplasmic translocation of HuR, which in turn stabilizes IL‐6 mRNA and ultimately drives synovial cell proliferation [28]. In cancer, cytoplasmic HuR stabilizes oncogenic transcripts such as MYC and BCL2, thereby driving proliferation and metastasis [29, 30]. Similarly, in renal pathology, HuR translocation has been implicated in promoting renal cell proliferation [31].

Although TWEAK‐induced metabolic dysregulation and AMPK’s role in controlling proliferation are each established [32–34], the functional connection between these pathways in LN—particularly through HuR‐mediated posttranscriptional regulation—has not been thoroughly investigated. Interestingly, AMPK signaling is known to modulate HuR trafficking [35], suggesting potential crosstalk between energy‐sensing and mRNA stability control.

This study aims to elucidate the role of TWEAK in promoting MCs proliferation, decipher the molecular mechanisms underlying dysregulation of the TWEAK–AMPK–HuR axis, and provide a novel therapeutic rationale for targeting TWEAK signaling in LN.

## 2. Materials and Methods

### 2.1. Animals and Cells

Kidney tissues from MRL/lpr or MRL/MpJ mice were previously retained by our laboratory. Human glomerular MCs (HMCs) were purchased from Shanghai Mcellbank Biotechnology Co., Ltd. The cell culture system was a complete medium configured with 10% fetal bovine serum (Corning, USA), 1% penicillin–streptomycin double antibody (Beyotime, China), and 89% DMEM high glucose medium (Gibco, USA) and was maintained in a cell culture incubator (Thermo, USA) at 5% CO_2_ and 37°C.

### 2.2. Patients

In total, 15 LN patients and 15 healthy volunteers were recruited from the population attending the Second Affiliated Hospital of Soochow University (Suzhou, China) from March 2021 to March 2022. This study was approved by the Clinical Research Ethics Committee of the Second Affiliated Hospital of Soochow University (Approval Number JD‐LK2023047‐IR01), and written informed consent was obtained from each participant. Participant characteristics are summarized in Table 1. The peripheral blood of each subject was collected, and serum was separated, and the serum was mixed in equal quantities by group, divided, and frozen at −80°C for spare use.

**Table 1:** Clinical features for SLE patients.

Parameters	SLE	Healthy
No. of subjects	15	15
Sex (female [%])	13 (86.7%)	13 (86.7%)
Age (mean ± SEM [years])	27.6 ± 10.5	28.3 ± 8.9
SLEDAI	9.8 ±7.1	N/A
Disease duration (mean ± SEM [months])	6.1 ± 4.2	N/A
Treatments		
Untreated (*n* [%])	3 (20.0%)	N/A
Corticosteroids (*n* [%])	11 (73.3%)	N/A
Antimalarials (*n* [%])	12 (80.0%)	N/A
Immunosuppressants (cyclophosphamide, mycophenolate mofetil, and calcineurin inhibitors [*n* (%)])	10 (66.7%)	N/A

### 2.3. CCK‐8 Assay

Approximately 10^3^ cells were seeded in 96‐well plates (Corning, USA) with 100 μL of medium in each well. After cell attachment, each well was replaced with 100 μL of medium containing the appropriate stimulating factors. After reaching the predetermined time, each well was incubated with 10 μL CCK‐8 solution (APE × BIO, USA) for 2 h away from light. Absorbance at 450 nm was measured using ELISA (TECAN, Switzerland).

### 2.4. Lentiviral Transfection

Human RNF31 HOIL‐1‐interacting protein (HOIP) interfering lentivirus carrying puromycin resistance gene and negative control interfering lentivirus were commissioned to be constructed by Sangong Biotech (Shanghai) Co., Ltd. Transfected and screened cells according to the instructions. Transfection efficiency was analyzed by western blot.

### 2.5. Western Blot

Total proteins were extracted using RIPA buffer (NCM, China) containing protease inhibitors (Beyotime, China) and phosphatase inhibitors (APE×BIO, USA). Nuclei and cytoplasmic proteins were extracted separately using the Nucleus and Cytoplasmic Protein Extraction Kit (Beyotime, China). Proteins were separated by SDS–PAGE and transferred to PVDF membranes (Millipore, USA). The membranes were closed with 5% milk for 1 h at ambient temperature, followed by overnight incubation with primary antibodies at 4°C. The primary antibodies used are as follows: anti‐HOIP (1:3000; CST, USA), anti‐AMPKα (1:1000; CST, USA), antiphosphorylated AMPKα (p‐AMPKα) (1:1000; CST, USA), anti‐HuR (1:1000; CST, USA), anti‐TWEAK (1:1000; CST, USA), anti‐Histone H3 (1:1000; Abcam, USA), anti‐GAPDH (1:10000; ProteintechGroup, China), anti‐β‐actin (1:3000; ProteintechGroup, China), anti‐Cyclin D1 (1:5000; ProteintechGroup, China), anti‐Bax (1:8000; ProteintechGroup, China), anti‐Bcl‐2 (1:4000; ProteintechGroup, China), and anti‐Met1‐Ub (1:1000; Sigma, USA). After washing with TBST, the membranes were incubated with goat anti‐rabbit IgG secondary antibody (1:3000, MULTI SCIENCES, China) for 2 h at ambient temperature. Finally, signals were detected using an ultrasensitive ECL chemiluminescence kit (NCM, China) and a chemiluminescence imaging system (Syngene, UK).

### 2.6. Coimmunoprecipitation

IP samples were prepared using an immunoprecipitation kit (Sangong, China). Cells were continuously lysed with lysis buffer at low temperature for 30 min. Subsequently, centrifuge at 12,000 rpm for 5 min and collect the supernatant. Next, the supernatants were incubated with anti‐AMPKα antibody or normal rabbit IgG at 4°C overnight. Cell lysates were then combined with Protein A/G plus‐Agarose overnight at 4°C. The collected precipitates were washed repeatedly with ice IP buffer and then centrifuged to remove the buffer, mixed well with loading buffer, sealed, and heated at 95°C for 5 min. Finally, the supernatant was analyzed by western blot.

### 2.7. Flow Cytometric Analysis

Cultured and stimulated cells in 6‐well plates (Corning, USA) at an appropriate density. Then, cells were collected, and cell suspensions were prepared using the Apoptosis Detection Kit (BD, USA) according to the instructions. Apoptosis levels were detected by flow cytometry (Beckman, USA).

### 2.8. Immunofluorescence

Paraffin sections or cell crawls were prepared in the usual way. The specimens were infiltrated with 0.1% Triton‐X 100 (Solarbio, China) for 20 min after PBS washing. Next, the specimens were blocked with 10% goat serum (Solarbio, China) at 37°C for 30 min and incubated with primary antibodies at 4°C overnight. After washing with PBS, the specimens were incubated with fluorescently labeled goat anti‐rabbit IgG secondary antibody (1:500; Abbkine, China) for 1 h at room temperature and protected from light. Antibodies used are as follows: anti‐Ki67 (1:500; Abcam, USA), anti‐HuR (1:500; CST, USA), DyLight 594 fluorescently labeled goat anti‐rabbit IgG secondary antibody (1:500; Abbkine, China), and FITC fluorescently labeled goat anti‐mouse IgG secondary antibody (1:500; Abbkine, China). PBS was used to wash the secondary antibody, and then the appropriate amount of DAPI‐containing antifluorescence quencher (Invitrogen, USA) was added. Immunofluorescence photographs were taken using a laser confocal microscope (Zeiss, Germany).

### 2.9. Statistical Analysis

All data are from three independent replicated experiments. For protein bands, grayscale values were analyzed using ImageJ. Statistical analyses were performed using GraphPad Prism 9. For data that conformed to a normal distribution, two‐by‐two comparisons were performed using *t*‐tests, and one‐way ANOVA followed by Tukey’s post hoc test was used for comparisons of multiple groups of data. Nonparametric tests were used for nonnormally distributed data. *p* *<*0.05 was considered a statistically significant difference.

## 3. Results

### 3.1. TWEAK Induces Hyperproliferation of MCs in LN

To investigate the mechanism of MCs’ hyperproliferation in LN, HMCs were stimulated with serum from LN patients. CCK‐8 assay results showed that LN patient serum significantly induced HMCs proliferation, with the most pronounced pro‐proliferative effect observed at a concentration of 15% (Figure 1A). Western blot analysis revealed that TWEAK protein expression was significantly upregulated in HMCs following stimulation with LN patient serum compared to healthy volunteers (Figure 1B). These findings suggest a strong association between renal cell proliferation and TWEAK in LN.

Figure 1TWEAK induces hyperproliferation of mesangial cells in lupus nephritis. (A) CCK‐8 assay demonstrated changes in the proliferative capacity of HMCs stimulated by LN serum. (B) Determination of TWEAK protein expression levels after serum stimulation in healthy volunteers or LN patients by western blot. (C) Ki‐67 expression levels in mouse kidney tissues measured by immunofluorescence. (D) Cyclin D1 expression levels in mouse kidney tissues determined by western blot. (E) Changes in proliferation levels of HMCs stimulated by rhTWEAK. (F) HMCs apoptosis levels after rhTWEAK stimulation determined by flow cytometry. (G) Bax and Bcl‐2 protein expression levels of HMCs stimulated by rhTWEAK. The results are expressed as mean ± SD; ^ns^ *p* *>*0.05,  ^∗^ *p* *<*0.05,  ^∗∗^ *p*  < 0.01,  ^∗∗∗^ *p*  < 0.001,  ^∗∗∗∗^ *p*  < 0.0001.

**Figure:**
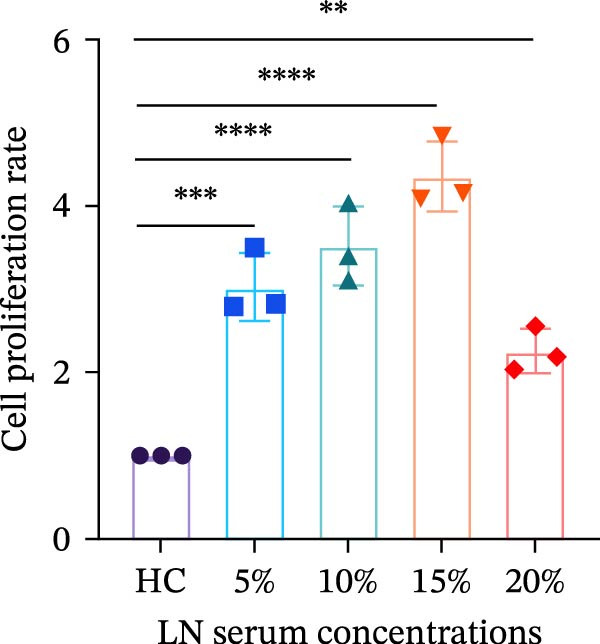
(A)

**Figure:**
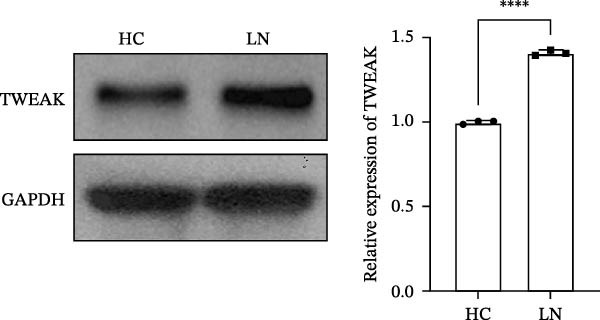
(B)

**Figure:**
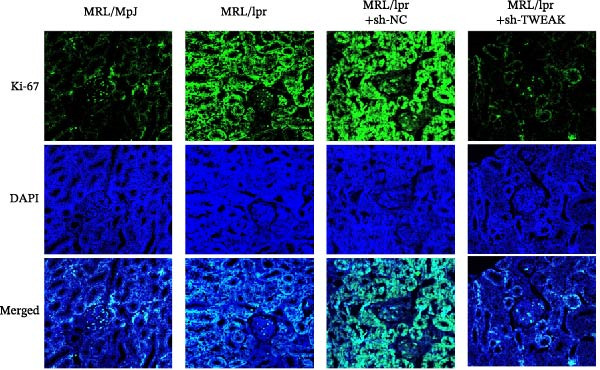
(C)

**Figure:**
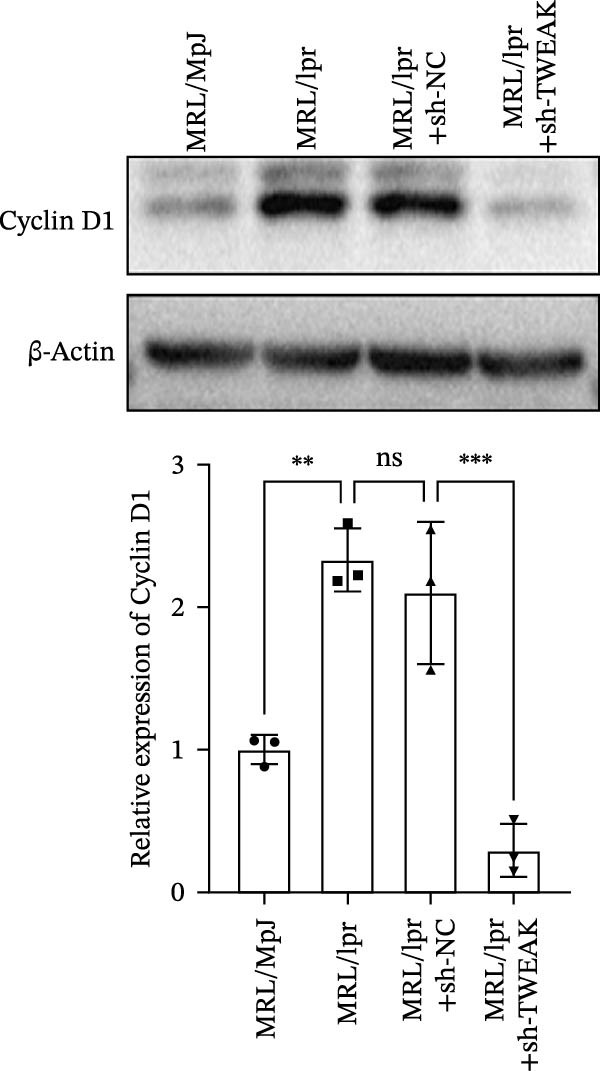
(D)

**Figure:**
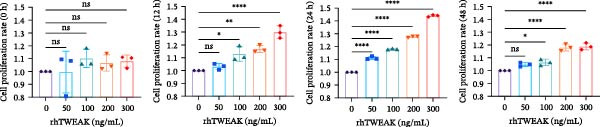
(E)

**Figure:**
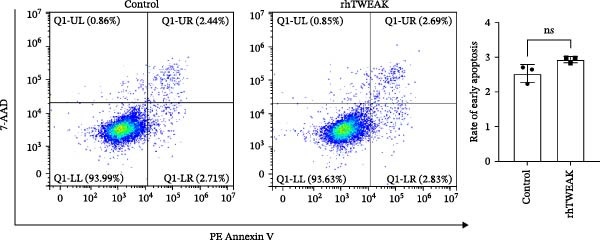
(F)

**Figure:**
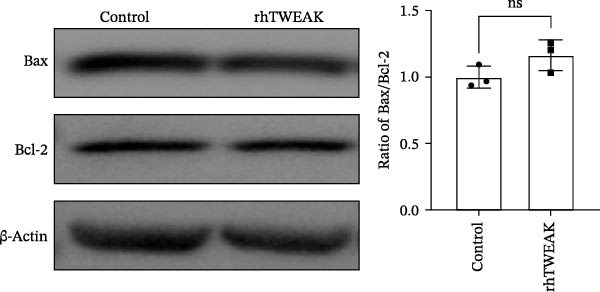
(G)

These were validated through animal experiments. Kidney tissues from MRL/lpr mice exhibited significantly higher expression of the proliferation marker Ki‐67 compared to control MRL/MpJ mice. Notably, TWEAK knockdown via RNAi significantly suppressed Ki‐67 expression in renal tissues (Figure 1C). The expression pattern of cell cycle protein Cyclin D1 mirrored that of Ki‐67 (Figure 1D), further confirming TWEAK’s role in driving cell cycle progression.

To directly demonstrate TWEAK’s pro‐proliferative effect, HMCs were stimulated with varying concentrations of recombinant human TWEAK protein (rhTWEAK). Results indicated that 100, 200, and 300 ng/mL of rhTWEAK promoted HMCs proliferation after 12 h of stimulation, with peak proliferative capacity reached at 24 h, which subsequently declined by 48 h (Figure 1E). Based on these findings, subsequent experiments utilized 200 ng/mL rhTWEAK stimulation for 24 h as the standard condition.

We next assessed the effect of TWEAK on MCs apoptosis. Flow cytometric analysis and western blot indicated that rhTWEAK had minimal impact on the expression ratios of Bax to Bcl‐2, suggesting no substantial role for TWEAK in inducing HMCs apoptosis under these experimental conditions (Figure 1F,G).

In conclusion, TWEAK plays a pivotal role in inducing MCs hyperproliferation in LN.

### 3.2. TWEAK Inactivates AMPK and Promotes HuR Nuclear‐Cytoplasmic Shuttling

Given the documented role of TWEAK in regulating energy metabolism pathways, we investigated whether TWEAK influences AMPK activity. Western blot analysis showed that rhTWEAK stimulation significantly reduced the levels of p‐AMPKα, indicating AMPK inactivation (Figure 2A).

Figure 2TWEAK inactivates AMPK and promotes HuR nuclear‐cytoplasmic shuttling. (A) Levels of AMPKα phosphorylation after rhTWEAK stimulation. (B) Immunofluorescent detection of subcellular localization of HuR. (C) The expression levels of HuR protein, from left to right, in the cytoplasm, nucleus, and total protein. (D) The expression levels of Cyclin D1 protein after rhTWEAK stimulation. The results are expressed as mean ± SD; ^ns^ *p* *>*0.05,  ^∗^ *p* *<*0.05,  ^∗∗∗^ *p*  < 0.001, and  ^∗∗∗∗^ *p*  < 0.0001.

**Figure:**
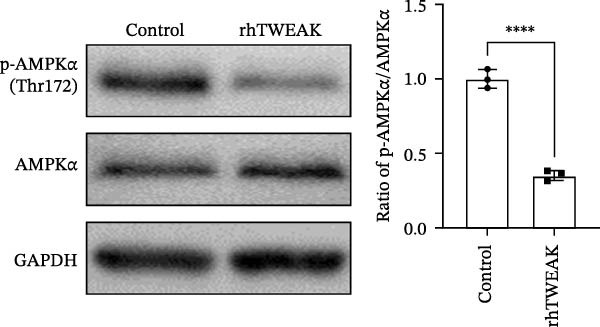
(A)

**Figure:**
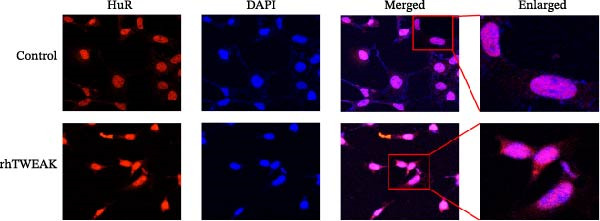
(B)

**Figure:**
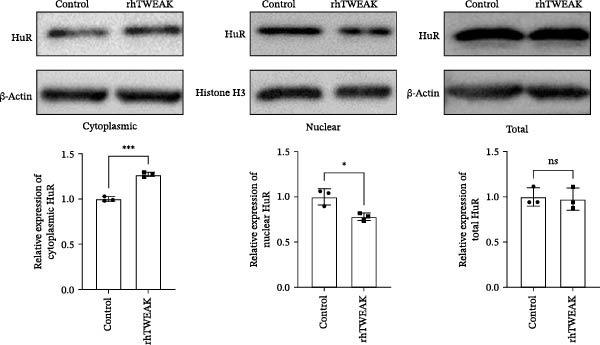
(C)

**Figure:**
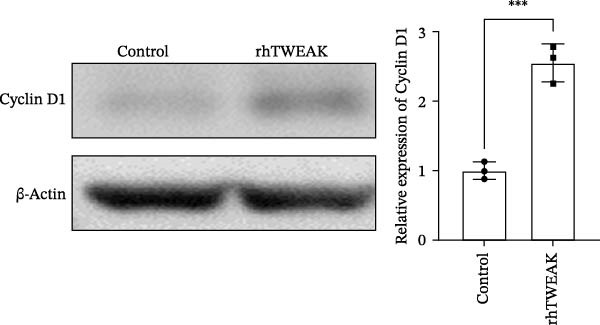
(D)

The nuclear‐cytoplasmic shuttling of HuR represents a fundamental regulatory mechanism governing its ability to modulate downstream target genes and cellular functions. To investigate this process, we assessed the subcellular localization of HuR, an RNA‐binding protein, by immunofluorescence. Our results revealed that rhTWEAK treatment promoted substantial nuclear‐to‐cytoplasmic translocation of HuR (Figure 2B). This observation was further supported by cellular fractionation assays, which confirmed a marked increase in cytoplasmic HuR protein levels accompanied by a corresponding decrease in nuclear HuR upon rhTWEAK stimulation, with no alteration in total HuR expression (Figure 2C).

### 3.3. HuR Nuclear‐Cytoplasmic Shuttling Drives Cell Cycle Progression and MCs Proliferation

To establish the pivotal role of HuR cytoplasmic translocation in TWEAK’s pro‐proliferative effects, we examined the expression of core cell cycle protein Cyclin D1. Results demonstrated that Cyclin D1 expression was low under resting conditions but significantly upregulated following rhTWEAK stimulation (Figure 2D). This pattern correlated strongly with observed HuR cytoplasmic translocation (Figure 2B,C) and cellular proliferation results (Figures 1E and 3C).

Figure 3HOIP‐mediated linear ubiquitination of AMPK contributes to TWEAK‐induced MCs hyperproliferation. (A) AMPK linear ubiquitination levels in each group, from left to right, are negative control IgG, positive control Input, and immunoprecipitation IP. (B) Validation of HOIP knockdown efficiency at the protein level. (C) HMCs proliferation levels under rhTWEAK stimulation after HOIP knockdown by CCK‐8 assay. (D) The expression levels of Cyclin D1 protein under rhTWEAK stimulation after HOIP knockdown. The results are expressed as mean ± SD; ^ns^ *p* *>*0.05,  ^∗^ *p* *<*0.05, and  ^∗∗∗∗^ *p*  < 0.0001.

**Figure:**
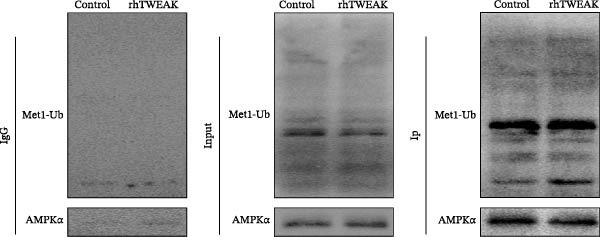
(A)

**Figure:**
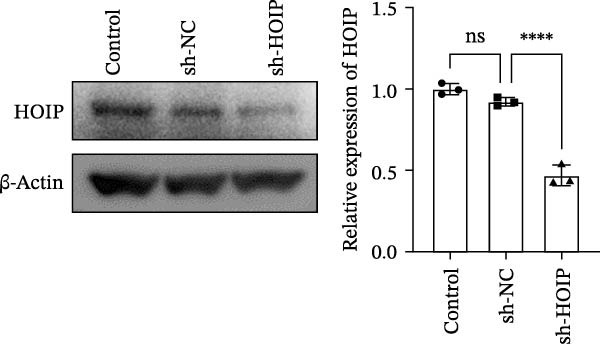
(B)

**Figure:**
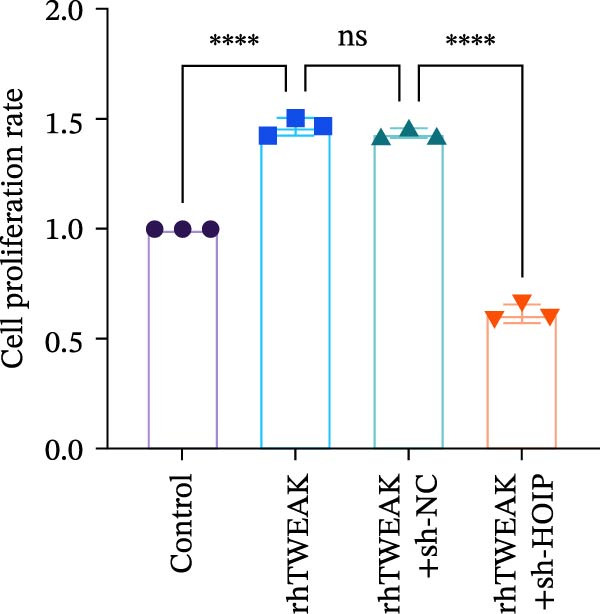
(C)

**Figure:**
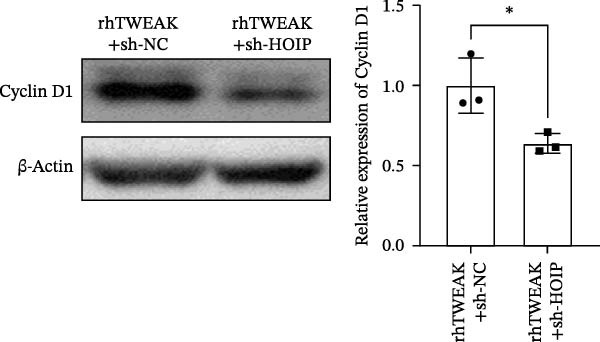
(D)

These results confirm that TWEAK‐induced HuR nuclear‐cytoplasmic shuttling serves as a critical nexus connecting upstream signaling with cell cycle regulation: cytoplasmic HuR stabilizes numerous pro‐proliferative mRNA transcripts, including Cyclin D1, thereby accelerating G1/S phase transition and ultimately driving MCs hyperproliferation.

### 3.4. HOIP‐Mediated Linear Ubiquitination of AMPK Contributes to TWEAK‐Induced MCs Hyperproliferation

In HMCs treated with rhTWEAK, the level of p‐AMPKα decreased, while total AMPKα expression remained largely unchanged. Ubiquitination, a critical posttranslational modification that regulates protein function, is increasingly recognized as a precise modulator of AMPK phosphorylation status—offering new perspectives on the regulation of AMPK activity. Notably, linear ubiquitination of LKB1, a key upstream regulator of AMPK, serves as an essential molecular trigger that activates LKB1’s kinase function, leading to the phosphorylation and activation of the AMPK pathway [21]. We therefore hypothesized that linear ubiquitination might mediate the TWEAK‐induced regulation of AMPK phosphorylation.

To test this hypothesis, coimmunoprecipitation assays were first performed, which confirmed endogenous interaction between AMPK and linear ubiquitin chains in HMCs. Further experiments revealed that stimulation with rhTWEAK significantly enhanced AMPK linear ubiquitination compared with controls (Figure 3A), suggesting that TWEAK‐induced linear ubiquitination may inhibit AMPK phosphorylation and thereby inactivate the kinase.

To define the functional role of the linear ubiquitination assembly complex subunit HOIP, we established a stable HOIP‐knockdown HMCs line (sh‐HOIP) (Figure 3B). Proliferation assays demonstrated that HOIP knockdown markedly attenuated the rhTWEAK‐induced increase in cell proliferation (Figure 3C) and the upregulation of Cyclin D1 (Figure 3D). Collectively, these results indicate that HOIP‐mediated linear ubiquitination of AMPK is necessary for TWEAK‐induced MCs hyperproliferation.

### 3.5. HOIP Knockdown Reverses TWEAK‐Induced AMPK Inactivation and Inhibits HuR Translocation

We further tested whether HOIP knockdown reverses TWEAK‐induced linear ubiquitination of AMPK and downstream signaling. Compared to sh‐NC controls, sh‐HOIP cells showed reduced AMPK linear ubiquitination following rhTWEAK stimulation (Figure 4A). HOIP knockdown also restored AMPK activity, as indicated by increased p‐AMPKα levels (Figure 4B).

Figure 4HOIP knockdown reverses TWEAK‐induced AMPK inactivation and inhibits HuR translocation. (A) AMPK linear ubiquitination levels in each group, from left to right, are negative control IgG, positive control Input, and immunoprecipitation IP. (B) Levels of AMPKα phosphorylation under rhTWEAK stimulation after HOIP knockdown. (C) Immunofluorescent detection of subcellular localization of HuR. (D) The expression levels of HuR protein, from left to right, in the cytoplasm, nucleus, and total protein. The results are expressed as mean ± SD; ^ns^ *p* *>*0.05,  ^∗^ *p* *<*0.05, and  ^∗∗∗∗^ *p*  < 0.0001.

**Figure:**
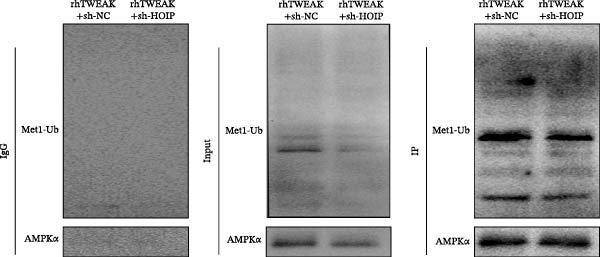
(A)

**Figure:**
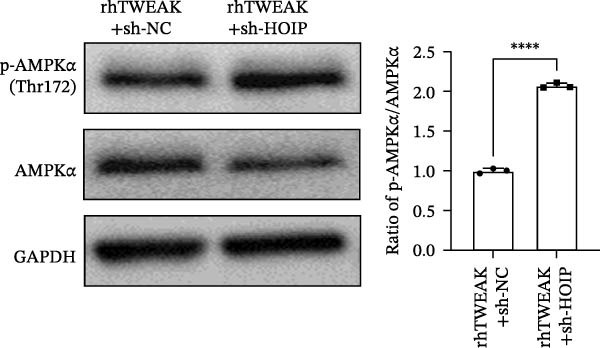
(B)

**Figure:**
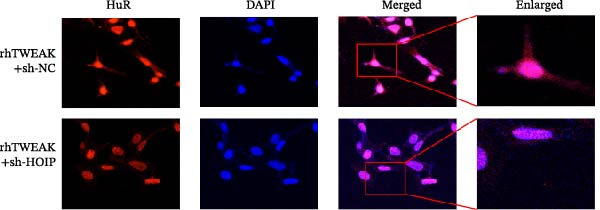
(C)

**Figure:**
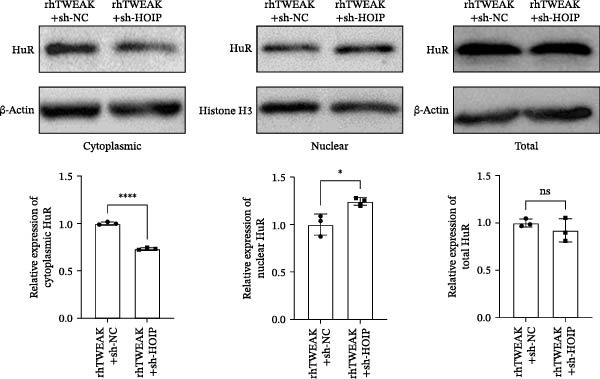
(D)

Immunofluorescence and cellular fractionation analyses demonstrated that HOIP knockdown markedly inhibited rhTWEAK‐induced HuR nuclear export (Figure 4C), leading to increased nuclear retention and reduced cytoplasmic levels of HuR, without altering total HuR expression (Figure 4D).

In summary, TWEAK promotes linear ubiquitination of AMPK via HOIP, resulting in AMPK inactivation and HuR nucleocytoplasmic shuttling. HOIP knockdown reverses these effects, restoring AMPK activity and nuclear retention of HuR.

## 4. Discussion

Abnormal proliferation of MCs plays a key role in the pathogenesis of LN and is currently a hotspot of LN research [36]. In this study, we observed that stimulation with serum from LN patients significantly enhanced the proliferative capacity of HMCs. Previous studies have indicated elevated serum levels of TWEAK in LN patients, which positively correlate with disease activity, establishing TWEAK as a biomarker of LN [11]. Consistent with this, our results demonstrated marked upregulation of TWEAK expression in HMCs following stimulation with LN patient serum. Moreover, TWEAK deficiency in MRL/lpr mice effectively attenuated renal cell proliferation. We further confirmed the direct pro‐proliferative effect of recombinant TWEAK on HMCs, aligning with earlier reports [37]. To further examine the effect of TWEAK on HMCs apoptosis, we performed flow cytometry and evaluated the expression of the apoptosis regulators Bax and Bcl‐2. rhTWEAK alone did not affect HMCs apoptosis or Bax/Bcl‐2 ratios; these results indicate that TWEAK primarily regulates proliferation rather than apoptosis and that its effects are independent of the Bax/Bcl‐2 pathway.

Most studies on TWEAK‐induced proliferation have focused on canonical pathways like NF‐κB. But accumulating evidence suggests the involvement of additional regulatory mechanisms [6]. Our study demonstrated that TWEAK treatment leads to a significant reduction in AMPK phosphorylation (a key indicator of AMPK activation) in HMCs. AMPK, a well‐characterized cellular energy sensor, modulates cell proliferation (e.g., by regulating oxidative stress in high glucose‐stimulated murine MCs) [38]. Although emerging studies have documented TWEAK‐mediated regulation of AMPK activity across various biological contexts [34, 39], the specific role of this TWEAK‐AMPK axis in governing the proliferation of HMCs remains poorly defined.

AMPK phosphorylation is regulated through multiple mechanisms, including allosteric modulation, upstream kinase signaling, and various ubiquitination modifications [20, 40]. While K63‐linked ubiquitination inhibits AMPK phosphorylation and K48‐linked ubiquitination induces degradation [22, 41, 42]. As a newly discovered form of ubiquitination modification, linear ubiquitination not only participates in regulating the progression of autoimmune diseases but also serves as a key regulator controlling cell cycle progression [43–45]. In this study, our coimmunoprecipitation assays identified linear ubiquitination as a novel regulatory mechanism: it suppresses AMPK phosphorylation (without inducing degradation) to inactivate the kinase.

Linear ubiquitination (a newly identified posttranslational modification) contributes to autoimmune diseases [46–48]. The attenuation of TWEAK‐induced mesangial cells proliferation following HOIP knockdown further supports the functional involvement of HOIP in mediating TWEAK‐driven proliferative responses, highlighting this pathway as a potential therapeutic target in LN, though exact regulatory mechanisms require further clarification.

We hypothesize that linear ubiquitination inhibits AMPK phosphorylation through three mechanisms: (1) steric hindrance, where ubiquitin chains block kinases from binding to Thr172 (threonine 172), (2) allosteric inhibition, where conformational changes lock the activation loop in a closed state, and (3) phosphatase recruitment, where ubiquitin chains recruit phosphatases to enhance local dephosphorylation and thus reduce net phosphorylation, and future studies will verify these mechanisms to clarify the basis of AMPK inhibition by linear ubiquitination.

In this study, TWEAK stimulation significantly upregulated Cyclin D1 protein expression, which coincided with cytoplasmic accumulation of HuR. Furthermore, TWEAK‐induced AMPK inactivation promoted nuclear export of HuR and enhanced cell proliferation. Cyclin D1, a core cell cycle regulator belonging to the G1 cyclin family [49, 50], the 3^′^ untranslated region (3’UTR) of its mRNA contains AREs,which serve as the core binding targets of HuR [51], it primarily facilitates the G_1_‐to‐S phase transition and plays a pivotal role in regulating cell proliferation, differentiation, and tissue homeostasis [52]. These results are consistent with previous reports indicating that AMPK inactivation promotes HuR cytoplasmic translocation in keratinocytes [53], while AMPK activation inhibits HuR shuttling in fibroblasts [54]. Notably, TWEAK did not alter total HuR expression but specifically affected its subcellular localization—a finding consistent with studies showing that HuR deficiency does not impair myocardial function [55]. This reinforces the conclusion that HuR translocation mediates TWEAK‐induced proliferation in HMCs.

The essential role of HuR shuttling is further corroborated by interventional experiments: HOIP knockdown prevented HuR nuclear export, concurrently reduced Cyclin D1 expression, and attenuated cell proliferation. These results establish a direct causal link between HuR localization, Cyclin D1 levels, and proliferative outcomes. Importantly, this regulation occurs posttranscriptionally without changes in total HuR abundance, underscoring the precision with which TWEAK modulates cell cycle progression. Collectively, these findings identify HuR‐mediated regulation of Cyclin D1 as a key mechanism in LN pathogenesis, wherein excessive MCs proliferation contributes to renal damage.

However, several limitations of this study should be acknowledged. First, the role of HOIP has not been directly validated in vivo, largely due to its essential function as the catalytic core of the linear ubiquitin chain assembly complex during embryonic development. Previous reports indicate that complete HOIP knockout leads to early embryonic lethality in mice, and patients with mutations in the LUBAC catalytic subunit HOIP present with autoinflammation combined with immunodeficiency [56–59], posing significant challenges for establishing constitutive HOIP‐deficient animal models suitable for chronic disease studies. Consequently, our findings are currently confined to the cellular level, elucidating the TWEAK/HOIP/AMPK/HuR signaling axis in vitro. Moving forward, conditional knockout models or kidney‐targeted delivery systems could be employed to selectively inhibit HOIP in relevant cell types or tissues, which would help clarify its precise role in LN pathogenesis in vivo—a key direction for future research. Second, while functional data and published literature [26, 27, 60] strongly support HuR‐mediated stabilization of Cyclin D1 mRNA in our experimental system, direct evidence of a physical interaction between HuR and Cyclin D1 mRNA—for example, via RIP or CLIP assays—remains to be obtained. Thus, our interpretation regarding this mechanism is based on the established involvement of HuR in posttranscriptional regulation of cell cycle‐related genes, and further validation will be important in subsequent studies. Third, the human serum samples analyzed here were collected from a relatively limited cohort. Although a clear trend was observed, studies with larger sample sizes will be necessary to strengthen statistical confidence and improve the generalizability of these preliminary findings.

In summary, our study identifies a novel TWEAK/HOIP/AMPK/HuR signaling axis critical for promoting mesangial cells proliferation in LN. We demonstrate that TWEAK induces linear ubiquitination of AMPK via upregulation of HOIP, leading to AMPK inactivation. This in turn triggers HuR nuclear‐cytoplasmic shuttling, ultimately driving cellular proliferation. Targeting this pathway may offer a promising therapeutic strategy for mitigating renal pathology in LN.

## Funding

This research was funded by the Suzhou Medical and Health Science Technology Innovation Project (Grant SKY2022146), the Suzhou Science and Technology Bureau Clinical Trial Institution Capability Improvement Project (Grant SLT2023035), and the Jiangsu Provincial Health Key Research and Development Project (Grant ZD2022032).

## Ethics Statement

This study complies with the Declaration of Helsinki; all experiments are conducted with full compliance with local, national, ethical, and regulatory principles and local licensing regulations. The Clinical Research Ethics Committee of the Second Affiliated Hospital of Soochow University approved this study. Each patient and healthy volunteer provided written informed consent.

## Conflicts of Interest

The authors declare no conflicts of interest.

## Data Availability

The datasets used and/or analyzed during the current study are available from the corresponding author upon reasonable request.
